# NEITHER HERE NOR THERE, YOU BECOME A STRANGER FOR BOTH SIDES: SUCCESSFUL AGING IN CHINESE AMERICAN OLDER ADULTS

**DOI:** 10.1093/geroni/igac059.2871

**Published:** 2022-12-20

**Authors:** Mushira Khan, Chien-Ching Li, Ezer Kang

**Affiliations:** Mather Institute, Evanston, Illinois, United States; Rush University, Chicago, Illinois, United States; Howard University, Washington, District of Columbia, United States

## Abstract

Previous studies have critiqued the inordinate emphasis on high physical and cognitive functioning and a relative absence of cultural factors in our understanding of the aging process. The multi-year Perceptions of Aging Well in Diverse Populations study explores (1) the meanings attached to aging well across cultures and (2) similarities and differences in these perceptions within diverse racial and/or ethnic groups. This presentation highlights findings from in-depth, semi-structured qualitative interviews with Chinese Americans 50 years and older (n=25; 10 male, 15 female; mean age 58.3 years). Thematic analysis of the data showed that generation status, age at arrival, density of social networks, and level of acculturation were key determinants of successful aging. Specifically, those who were born in the United States or had immigrated at an early age appeared to value their independence and were actively making retirement plans. Late-life immigrants were often lonely and more dependent on their adult children. Financial security and healthcare access were important considerations for mid-life immigrants. Several participants felt like an ‘outsider’ in the US but also felt like they were regarded as ‘foreigners’ in their country of origin, resulting in feelings of ambivalence and alienation. Many participants, particularly women, reported feeling less safe in their neighborhoods given the increasing prevalence of violence against Asian Americans in recent years and some were even contemplating moving to another country post-retirement. These findings provide insights into how aging is experienced among Chinese Americans and may help inform initiatives to support successful aging in this population sub-group.

